# External exposure assessment in the Fukushima accident area for governmental policy planning in Japan: part 1. Methodologies for personal dosimetry applied after the accident

**DOI:** 10.1093/jrr/rrac079

**Published:** 2022-12-12

**Authors:** Yukihisa Sanada, Kazuya Yoshimura, Rina Sato, Mariko Nakayama, Masaharu Tsubokura

**Affiliations:** Sector of Fukushima Research and Development, Japan Atomic Energy Agency, Fukushima, Japan; Sector of Fukushima Research and Development, Japan Atomic Energy Agency, Fukushima, Japan; Hitachi Solutions East Japan, Ltd., Miyagi, Japan; PESCO Co., Ltd., Ibaraki, Japan; Department of Radiation Health Management, Fukushima Medical University, Fukushima, Japan

**Keywords:** governmental policy, Fukushima Daiichi Nuclear Power Plant (FDNPP) accident, external exposure assessment, difficult-to-return areas, simulation

## Abstract

The evacuation order areas established due to the accident at the Tokyo Electric Power Company Holdings’ (TEPCO) Fukushima Daiichi Nuclear Power Plant (FDNPP) have been reorganized according to the decrease in ambient dose rates and the decontamination progress. The Japanese government decided to decontaminate the difficult-to-return areas and lift the evacuation order by 2030. This radiation protection strategy can be optimized by examining emergency exposure situations to date and the existing exposure after the accident. This article reviews the methods that can determine the individual radiation doses of residents who should return to their homes when the evacuation order is lifted in the specific reconstruction reproduction base area and the difficult-to-return areas outside this base area and summarizes the points to be considered when implementing these methods. In Part 1 of this article, we review the efforts made by the Japanese government and research institutes to assess radiation doses to residents after the FDNPP accident.

## INTRODUCTION

**Fig. 1:**
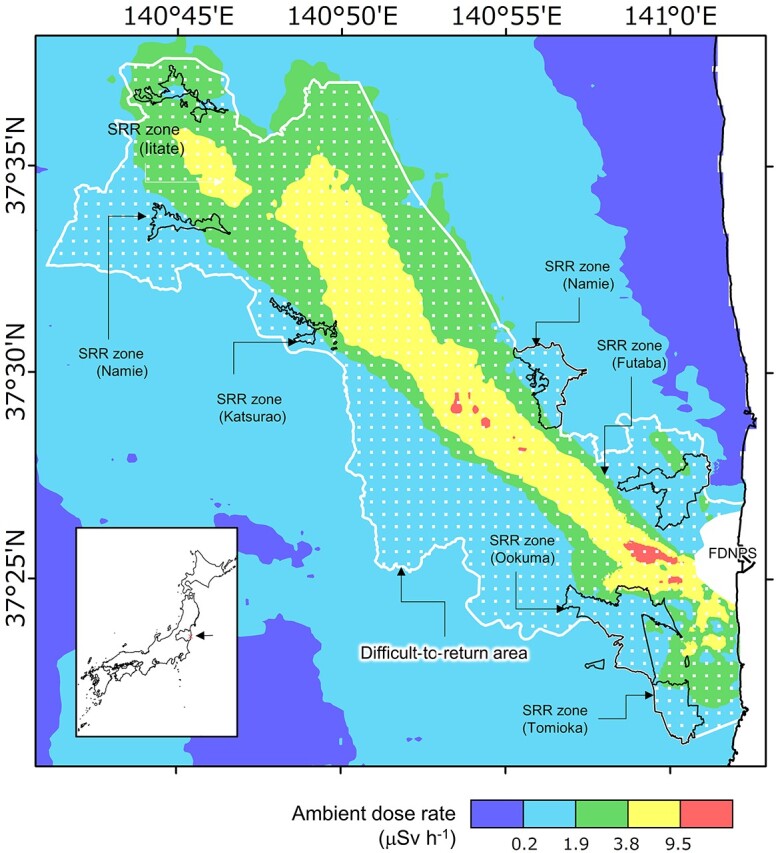
The specific reconstruction reproduction base area. The background map is of ambient dose rates in FY2020 [1].

The evacuation zones established due to the Fukushima Daiichi Nuclear Power Plant (FDNPP) accident at the Tokyo Electric Power Company Holdings (TEPCO) have been reorganized according to the decrease in ambient dose rates and the decontamination progress. In 2017, the local government designated a *specific reconstruction reproduction base area* (SRR zone) to accelerate the recovery and revitalization of the difficult-to-return areas as shown in Fig. 1 [1]. The central and local governments are working together to create an environment conducive for residents to return to their homes and lift the SRR zone between 2022 and the spring of 2023. In response to requests from local governments and recommendations from the ruling party, it was decided at the joint meeting of the 30th Reconstruction Promotion Council and the 55th Nuclear Emergency Response Headquarters held in August 2021 that decontamination and the removal of evacuation orders will be conducted in the 2020s to return to the areas.

Individual radiation doses are being monitored and controlled in the areas affected by the FDNPP accident in Fukushima Prefecture based on the *Basic Approach to Safety and Security Measures for Return to Home (For Specific Implementation of Protective Measures Based on Dose Levels)* (Nuclear Regulation Commission, November 2013). Furthermore, this approach states that, as part of efforts toward residents returning, the individual radiation doses assumed for residents after their return should be determined to help them make decisions. The article states that it is crucial to understand that personal exposure doses are measured primarily for residents living in areas after lifting the difficult-to-return areas and for residents who temporarily enter the difficult-to-return areas. Residents should also know their exposure doses, and local governments should know these levels so that residents can be aware of the health effects of radiation. The results have been used to help reduce anxiety.

A decade after the accident, the current situation around the FDNPP has entered the stage of an optimization process to be implemented in the post-accident recovery situation from the emergency exposure condition, as defined in the International Commission on Radiological Protection (ICRP) publication 111 [2]. In optimizing such protective strategies, it is necessary to scrutinize past emergencies and existing exposure conditions after the accident. However, the problem is far from being investigated comprehensively and holistically because the accident’s social impact was significant.

Ideally, as recommended by the ICRP [3], a reference level should be established to optimize radiation protection for the population. For this purpose, actual measurement of exposure doses and simulations based on the measured results are indispensable. This article reviews the dose exposure assessment methods that can be applied to the future lifting of the difficult-to-return areas for the SRR zone and the difficult-to-return areas outside the SRR zone, in order to determine the individual radiation dose levels for residents who are expected to return before they return, and summarizes points to be considered when implementing the methods. In Part 1 of this article, we review the efforts made by the Japanese government and research institutes to assess radiation doses to residents after the FDNPP accident. The methods for determining the exposure doses of residents are largely divided into two categories: actual measurement using personal dosimeters and calculation simulation. Several methods are also being considered for computational simulation, which can be divided by the type of parameters based on actual measurements. A comprehensive overview of these methods will be summarized. In Part 2, based on the information summarized in Part 1, we will discuss issues and points to be considered when applying these methods for policy decisions in the future.

## OVERVIEW OF METHODOLOGY FOR INDIVIDUAL DOSE EVALUATION

### Unit of external dose exposure

In this study, *external dose exposure* is positioned as the primary theme because radioactive cesium (^134^Cs, ^137^Cs) primarily caused the effects of the FDNPP accident, and external dose exposure overwhelmingly exceeds *internal dose exposure* [4]. The quantities related to external exposure are based on physical quantities, such as absorbed doses in organs and tissues in the human body. These quantities are broadly classified into *protective quantities*, such as equivalent and effective doses, and *practical quantities*, such as ambient dose equivalents.

**Fig. 2:**
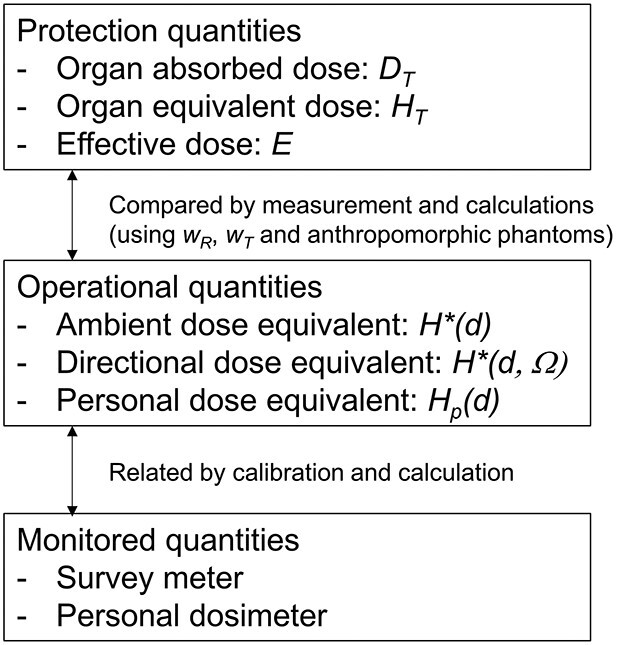
Relationship of quantities for radiological protection monitoring (modified by ICRP pub.74 Fig. 1 [5]).

Figure 2 shows the relationship between the quantities used to evaluate a dose to the human body from external exposure. The effective dose (*E)* is calculated by first multiplying each organ’s absorbed dose (*D_T_*) due to the radiation energy deposition by a radiation weighting factor reflecting biological effects depending on the radiation type and energy to obtain the equivalent dose (*H_T_*). This value is then multiplied by a tissue weighting factor that considers each organ’s radiation sensitivity to obtain the total body [5]. The results are added up and evaluated for the organs. Therefore, a single index can express the risk to the entire body from exposure to several types of radiation. However, since equivalent and effective doses cannot be measured directly because they are inside the human body, a practical dose was devised as a numerical value that can be measured. These practical quantities are measured as *monitored quantities* obtained using measuring instruments. The relationship between protective and effective doses has been tabulated in the ICRP publication 74 for each irradiation condition from a radiation source using calculation simulations and other methods [5].

From a radiation protection viewpoint, the effective dose (the amount of protection) is required as the individual exposure dose. The effective dose can be estimated using various methods; however, generally, it can be expressed using the following equation [6].

(1) \begin{equation*} \boldsymbol{E}=\sum \boldsymbol{c}\bullet \dot{\boldsymbol{D}}\left(\mathbf{p}\right)\bullet \boldsymbol{t}\left(\mathbf{p}\right), \end{equation*}

where *c* is the conversion factor from the measurable dose to the effective dose, *D ˙(p)* is the measurable dose rate at location *(p)*, and *t(p)* is the time spent at location *(p)*.

Since the effective dose cannot be measured directly, it is obtained by multiplying a measurable quantity by a conversion factor. Measurable doses are mainly personal dose equivalent and ambient dose equivalent. The effective dose for the period can be evaluated by summing up the above equations for the period to be evaluated. In this article, we review the methods for evaluating individual exposure doses and summarize the points to be considered in the evaluation, focusing on the ‘case of using personal dose equivalents measured with personal dosimeters’ and the ‘case of using ambient dose equivalents measured with survey meters, etc.’ as measurable doses.

Firstly, notation notes are shown below in the case of using personal dose equivalents measured with personal dosimeters. Since the situation of exposure in the environment is similar to rotational irradiation, the personal dose equivalent is close to the effective dose, which is the protective dose [5]. Therefore, as an approximation, the measurement results using personal dosimeters are often regarded as the individual exposure dose. In addition, since personal dose equivalents can be measured directly by wearing personal dosimeters in daily life, personal exposure doses can be obtained with relatively high accuracy. As shown in Fig. 2, ‘values measured by personal dosimeters’ are referred to as ‘personal dose equivalents’ in this article, although the two are strictly different.

Secondly, notation notes are shown below in case of using ambient dose equivalents measured with survey meters, etc. Although the ambient dose equivalent is larger than the effective dose regardless of irradiation conditions [5], the effective dose can be calculated from the measured ambient dose equivalent because the quantitative relationship between them is well organized as described above. The effective dose can be estimated with a certain degree of accuracy by taking into account the behavioral pattern of the person to be assessed, and is characterized by the fact that a realistic individual exposure dose can be obtained without long-term measurements using personal dosimeters. In this article, ‘values measured with survey meters, etc.’ will be referred to as ‘ambient dose rates’ for convenience. The value obtained by integrating the ambient dose rate with the time spent in the area will be referred to as the ‘ambient dose equivalent’.

This article refers to *values measured with survey meters* as *ambient dose rates* for convenience. The value obtained by integrating the ambient dose rate with the time spent in the area will be referred to as the *ambient dose equivalent*. This study uses *individual exposure dose* to include *personal dose equivalent* measured using personal dosimeters and *effective dose* estimated by simulation based on ambient dose rates measured using survey meters.

### Methodology classification

Assessment includes the *actual measurement* of personal dose equivalents using personal dosimeters and *simulation* using calculation codes. Several combinations of these methods have been applied in the areas affected by the FDNPP accident. The objectives of individual exposure dose assessments can be categorized by time axis and target. From a time axis viewpoint, the methods can be divided into *retrospective* assessment (to evaluate past individual exposure doses) and *predictive* assessment (to make policy decisions on decontamination and evacuation zones). Furthermore, the targets can be broadly classified into individual and population assessments. The most appropriate method should be applied according to the time axis and target intended in individual exposure dose assessments. Table 1 classifies typical methods for individual exposure dose assessments.

**Table 1:** Classification of individual exposure dose assessment Methods

Method	Time axis	Target	Application example	c	*D(p)*	*t(p)*
Measurement by Personal Dosimeter	Retroactive	Individual assessment	Grasp for individual exposure dose	Personal dose equivalent		Actual measurements
Combination of personal dosimeter measurements and simulation	Retroactive	Group assessment	Evaluate individual exposure dose levels in a region	Personal dose equivalent		Actual measurements or listening comprehension
Simulation based on ambient dose rates assuming individual lifestyle behavior	Predictive and retroactive	Individual assessment	Evaluate individual exposure dose to specific patterns of lifestyle behavior	Simulation-based coefficients	Ambient dose rate	Actual measurements or listening comprehension
Probabilistic simulation assuming distribution of each parameter such as group behavior	Predictive and retroactive	Group assessment	Evaluate the individual exposure dose level of a population in an area	Simulation-based coefficients	Ambient dose rate estimates based on initial deposition	Listening comprehension

An example of an actual measurement of personal dose equivalents using personal dosimeters is the project by the Cabinet Office to measure the personal dose equivalents of residents in each municipality for a certain period during a year as part of the Fukushima Restoration Acceleration Grant Project [7]. Although such individual dose assessment projects conducted by the government and local governments are primarily a service to individuals, some reports of studies have used Monte Carlo simulations to calculate one-year individual doses based on data from such a partial period and evaluate individual dose levels in a population [8].

However, simulations rather than actual measurements are used to evaluate individual exposure doses for a population. Simulations calculate individual exposure doses using parameters, such as the ambient dose rate and time spent at the location of residence, and reduction factors for indoor and outdoor ambient dose rates according to the daily behavioral patterns. Sato *et al.* (2019) interviewed residents living near FDNPP about their behavioral patterns and assessed their exposure doses according to their behavioral patterns [9]. Furthermore, Takahara *et al.* (2020) proposed a probabilistic method to assess individual exposure doses for a group by assuming a group’s behavioral patterns based on a questionnaire survey in Fukushima City [10]. The following and subsequent chapters present these methods’ application status and characteristics.

#### Measurement using a personal dosimeter

Measurement by personal dosimeter is a reliable method of assessing retroactive individual exposure dose, as shown in Table 1. On the other hand, personal dosimeters must be worn on the subject’s body surface throughout the day, making it very difficult to obtain accurate data.

**Fig. 3:**
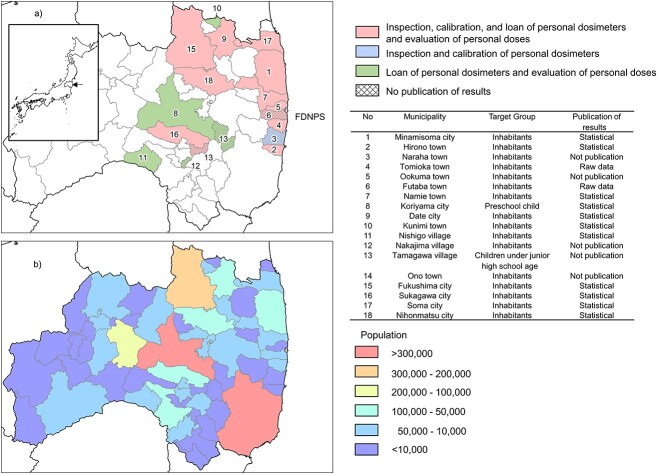
Municipalities and population of project implementation using the Fukushima Restoration Acceleration Grant Program.

After the FDNPP accident, many local governments, including Fukushima Prefecture, have been working to measure residents’ exposure doses. Many of these efforts have been conducted under the Fukushima Restoration Acceleration Grant Program [7], which is based on a service in which individual dosimeters are loaned out by each municipality to residents who wish to use them as shown in Fig. 3. This project combines the inspection and calibration of personal dosimeters to be lent to residents, and the lending of the dosimeters to residents, the reading out of measurement data, and the provision of information to individuals. Some municipalities only perform the inspection and calibration, while their staff members perform the lending and other private vendors. Since these projects are intended to provide services to individual residents who wish to use them, the results are generally not disclosed to the public, but some municipalities disclose the measurement results on their websites or in public relations magazines as statistical information. Fukushima City and Koriyama City, which have particularly large populations, are actively acquiring measured individual exposure doses data for their residents under this program. Iwaki City, the second most populous city in the coastal area, did not conduct actual measurements of individual exposure doses because of its low dose rate. These data have not been released in a form that would make them available to researchers due to privacy considerations. However, some statistical data analyzed by a conference body of experts in local governments are available to the public.

One large-scale government project was the *Project to ascertain individual radiation doses of residents in Fukushima Prefecture (Project to ascertain and manage individual radiation doses of residents in Fukushima Prefecture in 2014)* conducted by the Ministry of the Environment. In FY2014, personal dose equivalent data were obtained from 95 residents in Tamura City and Kawauchi Village using personal dosimeters (83 residents in Tamura City [average of 163 days per person] and 12 residents in Kawauchi Village [average of 81 days per person]) [11]. Furthermore, a technical review meeting with experts was held, and the *Guidelines for Measurement of Personal Exposure Doses of Residents after the Accident at TEPCO’s Fukushima Daiichi Nuclear Power Station and Handling of the Results* were formulated [12]. In FY2015, personal dose equivalent data were obtained from 59 residents in Tamura City (25, with an average of 177 days per person), Kawauchi Village (21, with an average of 226 days per person, and Naraha town (13) using personal dosimeters [13]. These projects are rare cases where individual dose equivalent measurements were conducted for more than 100 days for multiple individuals; however, the reports have not been disclosed on the Ministry of the Environment website since FY 2015.

Several academic analyses of individual dose assessments have been reported by individual researchers or in collaboration with local governments. Harada *et al.* (2014) have comprehensively assessed the risk of health effects by measuring actual external doses for 459 people in three lifted evacuation zones, as well as calculating inhalation and ingestion exposures [14]. Tsubokura *et al.* compared the external doses of 100 people in Minami Soma City, which became an evacuation zone immediately after the accident, and in three areas in Japan where the air dose rate of natural radiation is considered relatively high [15]. Murakami *et al.* (2019) reported individual dose equivalent data for more than 20 000 residents provided by Minamisoma City from 2013 to 2016 [16], and Naito *et al.* (2015) similarly analyzed the relationship between ambient dose rates from airborne radiological monitoring and individual dose equivalents by comparing ambient dose rate data over a wide area from airborne radiological monitoring [17]. Adachi *et al.* (2015) [18] and Nomura *et al.* (2016) [19] focused on pupils, and from a large amount of measured data, the former discussed the differences in high schools in other regions of Japan and Europe, whereas the latter determined individual dose equivalents at individual stay locations received in Minamisoma City. Uchiyama *et al.* (2020) compared personal dose equivalent data and GPS-based behavioral patterns of 300 TEPCO Holdings employees in Tokyo and Fukushima Prefecture. They found that annual personal doses were typically less than 1 mSv and that the personal doses received by outdoor workers were significantly higher [20]. Similarly, Sato *et al.* (2022) obtained data for 148 days from 36 people by combining personal dose equivalent data and GPS location data to verify the accuracy of a simulation that considers lifestyle behavior patterns, as described in the next chapter. They discussed the differences between indoor and outdoor locations [21]. Other cases can be found where the number of subjects was small, but where actual doses were measured over a long period of time [22], or where internal exposure doses assessed by measuring radon concentrations indoors were compared with external exposure doses [23].

These personal dosimeters measure individual dose equivalents to help reduce residents’ concerns about the health effects of radiation. Depending on the municipality, thousands to tens of thousands of residents are covered, and sufficient data have been obtained to examine the individual radiation doses for the entire population. Ongoing surveys also indicate decreasing individual radiation doses since the accident. However, some have highlighted problems from an academic perspective in the acquisition and analysis of individual dose equivalents and their comparison with ambient dose rates. These problems include the lack of considering the representativeness of data, the need for analysis that considers the characteristics of ambient dose rate data to be compared, and mismatches in analysis and statistical methods to be applied [24]. Even now, measurement optimization regarding interpreting the results is being attempted.

#### Simulation-based on ambient dose rates

In evaluating daily individual exposure doses, such as existing exposures, it is challenging to continuously measure residents’ dose equivalents throughout the year, unlike in the case of occupational dose exposure management. Therefore, cases have been reported where parameters related to individual exposure dose assessments are prepared in advance, and computational simulations are used. Simulation methods can be divided into three categories as shown in Table 1.

Firstly, combination of personal dosimeter measurements and simulation is a method to get an overall picture from computational simulation based on limited number of actual measurements and is suitable for the retroactive evaluation of group assessment. Tsubokura *et al.* (2019) evaluated the effect of decontamination on individual exposure doses based on statistical differences between the timing of decontamination and individual exposure doses in Minamisoma City [25]. The data were categorized by location and period in this evaluation to grasp the trend. Multiple regression analyses were performed to obtain the average reduction rate of individual exposure doses before and after decontamination. Based on the average value, the uncertainty of the individual exposure dose reduction rate was calculated by randomly changing variables using a Monte Carlo simulation. This approach is an example of combining measurements and simulations to determine the retrospective individual exposure dose for a population.

**Fig. 4:**
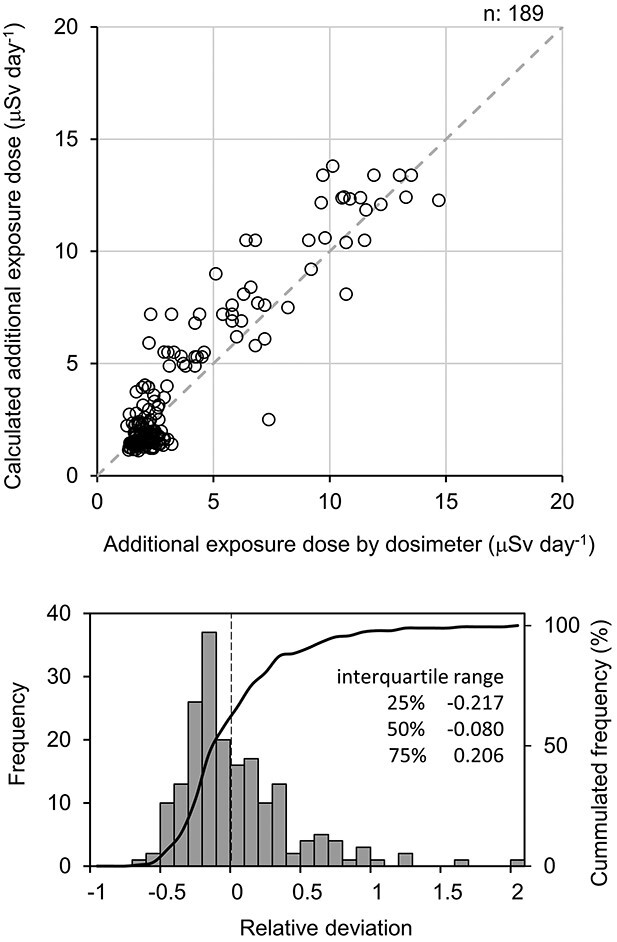
Example for comparison with measurements using a personal dosimeter and simulation based on ambient dose rates assuming individual lifestyle behavior. The authors revisited this figure with new data added to the data in Sato *et al.* 2022 [21].

Secondly, simulation based on ambient dose rates assuming individual lifestyle behavior is a method for evaluating individual exposure dose based on information on individual lifestyle behavior and ambient dose rates. The advantage of this method is that uncertainty can be evaluated by comparison with actual measurement by personal dosimeter. A typical example of this approach is the Fukushima health management survey which was conducted to reconstruct exposure doses immediately after the accident [26,27]. The method is based on the results of interviews of residents’ behaviour immediately after the accident and the results of a radiological radiation survey. The project is not only concerned with external exposure, but also with the health of a wide range of prefectural residents. In recent years, the accuracy of the method has been assessed by comparison with actual measurements. Sato *et al.* (2019) developed a method to calculate annual personal dose equivalents based on ambient dose rate information continuously measured by walking and driving surveys [9], location information and time spent in the area interviewed from residents, indoor and outdoor reduction factors, and conversion factors from ambient dose equivalent to effective dose. The accuracy of this method was evaluated by comparing it with information measured by personal dosimeters [21]. An example of the accuracy verification of this method obtained by the authors is shown in Fig. 4. This figure compares the results of daily additional exposure doses measured by personal dosimeters (*D_dos_*) and the results of daily additional exposure doses calculated from lifestyle behavior patterns and ambient dose rates (*D_cal_*) for local government officials and decontamination workers in Fukushima. In addition to the general scatter plots, the figures are also compared with histograms of relative deviations (*Rd*) calculated using the following formula.

(1a) \begin{equation*} Rd=\left({D}_{cal}-{D}_{dos}\right)/{D}_{dos} \end{equation*}

Such an accuracy assessment is essential information to be reflected in governmental policies. The Nuclear Regulation Authority adopted the developed method to evaluate the residents’ predictive exposure doses when the evacuation zone is lifted [1]. This method, defined as a *lifestyle behavior model*, is effective for predictive assessment, although its accuracy is lower than that of assessments based on actual measurements.

Then, in contrast to evaluation methods focusing on individuals, such as the ‘lifestyle behavior model,’ methods for evaluating individual radiation doses for groups such as municipalities have been reported. Takahara *et al.* (2020) devised a probabilistic method to evaluate individual exposure doses of a population using a Monte Carlo simulation by assuming each parameter’s distribution, such as the population’s behavior [10]. A method has been devised to evaluate a population’s exposure dose probabilistically. According to the ICRP recommendations [28], the concept of a *representative individual* is recommended to limit individual exposure. A representative individual is defined in the same report as *less than about 5% that a person drawn at random from the population will receive a greater dose*. This method’s simulation results help determine personal radiation protection policies that consider international recommendations. Thus, using individual exposure dose assessments by simulation allows predictive and population assessments. However, since the evaluation is performed using parameters, certain uncertainties exist, and their optimization is being attempted.

## CONCLUSION

Table 2 summarizes the characteristics of actual measurement methods and some simulations of individual exposure doses discussed so far. This review summarizes the assessment of individual radiation doses for the future lifting of evacuation orders in the SRR zone and the difficult-to-return areas outside the SRR zone as follows.

- While measurements using personal dosimeters are unsuitable for population and predictive assessments, they are suitable for providing individuals with realistic personal exposure doses. They should be performed under appropriate measurement and assessment conditions based on an understanding of the characteristics of the instruments used.
- While simulations of individual exposure doses are suitable for population and predictive assessment, there is a certain degree of uncertainty, and the parameters must be set appropriately based on past knowledge and actual measured values.

Part 1 of this review is divided into two chapters and summarizes the status of individual exposure dose assessment for residents in Japan after the FDNPP accident. To reflect the results in actual policies in the future, some individual points should be noted in the actual measurements and simulations of individual exposure doses. In part 2, issues of exposure dose assessment will be discussed based on the discussion in part 1.

**Table 2:** Combining objectives and methods in individual exposure dose assessments

	Objectives (categorized by time frame and target)			
	Retroactive		Anticipatory	
	Personal	Group	Personal	Group
Measurement by Personal Dosimeter	Suitable for the purpose	Applicable	Difficult to apply	Difficult to apply
Combination of personal dosimeter measurements and simulation	Applicable	Suitable for the purpose	Difficult to apply	Difficult to apply
Simulation based on ambient dose rates assuming individual lifestyle behavior	Suitable for the purpose	Applicable	Suitable for the purpose	Applicable
Probabilistic simulation assuming distribution of each parameter such as group behavior	Difficult to apply	Suitable for the purpose	Difficult to apply	Suitable for the purpose
